# When there is no justice, we need an old HERO. The trickle-down effect of psychological capital: the moderating role of organizational justice and leaders’ age

**DOI:** 10.3389/fpsyg.2024.1256721

**Published:** 2024-02-01

**Authors:** Milena Gojny-Zbierowska

**Affiliations:** Department of Entrepreneurship, University of Economics, Katowice, Poland

**Keywords:** psychological capital, perceived organizational justice, age, cross-over, trickledown, resilience

## Abstract

The objective of the study was to explore the impact of leaders’ ages and followers’ perceptions of organizational justice (POJ) on the transfer of psychological capital (PsyCap) from leaders to followers, particularly examining how employees’ resilience is influenced by leaders’ PsyCap. While some evidence exists regarding PsyCap’s trickle-down effect, the specific circumstances triggering this phenomenon remain unclear. This study investigates the relationship between followers’ and leaders’ PsyCap, employing the Social Cognitive Theory and considering the moderating effects of leaders’ age and POJ. The study focused on leader-follower dyads within a randomly selected sample of 406 businesses, encompassing 812 respondents. The survey investigation utilized the CAPI approach. The hypothesized model underwent testing through multilevel dyadic regression analysis, employing an actor-partner interdependence model. The findings support two moderators —employees’ POJ and managers’ age — and highlight the cross-over effect of PsyCap. Specifically, a stronger trickle-down link is observed when a leader is older and operating within a low POJ environment. Additionally, the study revealed a positive correlation between employees’ PsyCap and POJ. The development of POJ enhances employees’ PsyCap and resilience, while leaders’ PsyCap may compensate for organizational POJ deficiencies. This study is among the first to explore PsyCap’s moderators, specifically analyzing leaders’ ages and POJ as factors influencing the cross-over effect of PsyCap. By identifying previously unrecognized moderators affecting the cross-over PsyCap effect, this research contributes significantly to the PsyCap literature.

## 1 Introduction

Psychological capital is a positive psychological state encompassing personal resources such as hope, efficacy, resilience, and optimism (Luthans et al., 2007), forming the acronym HERO. Research has consistently shown its significant benefits for work-related outcomes, including performance, productivity (Peterson et al., 2011; Baykal and Zehir, 2018), engagement (Gao et al., 2023), innovative behavior (Karakitapoğlu-Aygün et al., 2020; Özsungur, 2020; Li et al., 2021), and well-being (Luthans et al., 2013; Youssef-Morgan and Luthans, 2015). Employees with higher PsyCap levels tend to exhibit be more satisfied, committed, relaxed (Avey et al., 2010, 2011b; Luthans and Youssef-Morgan, 2017) and openness to change (Liu, 2021). Individuals who possess hope, self-confidence, resilience, and optimism are valuable assets in any organization for various reasons. Given the ongoing global changes, the rise of artificial intelligence potentially replacing jobs, increased workplace pressures, the impact of events such as the COVID-19 pandemic and ongoing conflicts contributing to heightened feelings of danger, organizational contexts often encounter adverse events leading to feelings of threat and insecurity. These circumstances may explain the growing prevalence of anxiety disorders, depression, and reported psychological distress among individuals (Heitzman, 2020; Statistics Poland, and Social Surveys Department, 2020; Pierce et al., 2021). Mental health symptoms result in increased absenteeism and reduced employee productivity. For instance, it is estimated that psychological distress causes an AU$5.9 billion reduction in Australian employee productivity annually (Hilton et al., 2009). Notably, research from Northern Ireland suggests that any anxiety disorder accounts for 32.3% of all days out of role (Ennis et al., 2016), emphasizing the impact of mental health issues on workplace functionality. Therefor resilience defined as the ability to bounce back from crises and losses, is increasingly crucial in today’s World. This study’s primary motivation is to explore strategies for making resilience and PsyCap more sustainable within organizations, particularly investigating the transfer dynamics between leaders (supervisors) and followers (employees, subordinates). Trickle-down leadership refers to the relationship in which leaders’ behaviors and attitudes have a significant influence on their followers’ attitudes and behaviors (Chen et al., 2019; Tang et al., 2022). Research confirms that leaders’ behavior can impact employee psychological capital (e.g., Avey et al., 2010; Walumbwa et al., 2010; Chen et al., 2019). The internal connection between leader PsyCap and employee PsyCap aligns with the social cognitive theory, suggesting that individuals learn by observing and imitating the behaviors and attitudes of others (Bandura, 1977). Social cognitive processes such as learning and identification with the leader may explain why leaders with high PsyCap serve as role models for their followers, fostering similar positive psychological states (Avey et al., 2011a,b; King et al., 2015). Moreover, longitudinal and experimental studies support the idea that PsyCap development can occur through training interventions suggesting its learnability (Luthans et al., 2010; Demerouti et al., 2011; Dello Russo and Stoykowa, 2015). Resilience, on the other hand, might be also facilitated by exposure to potentially traumatic events and stressors (Eve and Kangas, 2015). Recent research indicates that moderate levels of adversity correlate with increased resilience (Seery et al., 2010).

Despite the acknowledged importance of PsyCap transfer, the understanding of its moderators remains limited. Research focusing on the relationship between leaders’ and employees’ PsyCap often emphasizes the lack of a contextual perspective and the need to investigate moderator variables (Story et al., 2013; Xu et al., 2017; Chen et al., 2019). Understanding the boundary conditions of the PsyCap trickle-down effect is pivotal for PsyCap development, contributing to a more positive and resilient workforce. This study aims to bridge this gap in the literature by investigating when the PsyCap cross-over effect is strongest and when the impact of leader PsyCap on followers’ resilience is most significant.

An analysis of the existing literature has identified potential moderating factors in this relationship, with leaders’ age and perceived organizational justice (POJ) (Moorman, 1991; James, 1993; Colquitt, 2001; Fox et al., 2001; Ambrose et al., 2002; Strack et al., 2008; Ambrose and Schminke, 2009; Verworn et al., 2009; Zhu et al., 2013; Lee et al., 2018; Khattak et al., 2019; Collins, 2021; Singh, 2021). Age has gained relevance in organisational research due to ageing workforces and age-related stereotyping affecting workers aged 55 and above (Radl, 2012; Bowman et al., 2017). Research indicates that age stereotypes negatively impact hiring opportunities for older workers (Fasbender and Wang, 2017) and lead to discrimination in training, performance appraisals, and mistreatment (Harris et al., 2018; Turek and Henkens, 2020). Leaders’ age might moderate the transfer of PsyCap from leader to employee, contributing to the ongoing debate surrounding age-related issues.

Perceived organizational justice (POJ), viewed as an employee-based resource, significantly shapes organizational behaviors (Lee et al., 2018; Collins, 2021). It refers to an employee’s perception of fairness in the workplace and their behavioral response to these perceptions. Previous research has demonstrated that organizational justice can moderate the relationship between leadership behavior and employee outcomes, buffering negative and amplifying positive effects (Ambrose and Schminke, 2009; Zhu et al., 2013). When employees perceive their leaders as fair and just, they are more likely to develop higher levels of psychological capital (Huang et al., 2020; Wang et al., 2020). Conversely, perceiving leaders as unfair or unjust can lead to negative emotions such as anger or frustration, which can decrease employees’ psychological capital (Li et al., 2019). Moreover, organizational justice perception can help address organizational problems related to employee turnover and absenteeism. When employees perceive their leaders as fair and just, they tend to be more satisfied with their jobs and committed to their organizations, resulting in decreased turnover and absenteeism rates (Greenberg, 1993; Eisenberger et al., 2002). Exploring how the relationship between leader and employee psychological capital is influenced by perceptions of organizational justice contributes to understanding how leadership behavior affects employee outcomes (Huang et al., 2020). This choice of organizational justice perception as a moderator variable has practical implications for mitigating the negative effects of leadership behavior on employee psychological capital, as well as theoretical significance in understanding the mechanisms behind the trickle-down effect. Understanding these factors’ roles contributes to a deeper comprehension of organizational challenges leadership’s impact on employee outcomes. Therefore, this study aims to investigate the influence of leaders’ age and employees’ POJ on the cross-over of psychological capital from leaders to followers. It builds upon previous research by examining organizational and managerial characteristics impacts on the transfer of hope, efficacy, resilience, and optimism. Analyzing these factors as the boundary conditions contributes to existing PsyCap literature and leadership studies. Moreover, this research offers a fresh perspective by highlighting how a leader’s age influences the learning and identification process with a supervisor. Contrary to the age-related debates, this study demonstrates that older age can facilitate the transfer of PsyCap. Drawing upon the social cognitive theory and conceptualizing PsyCap transfer mechanisms, this study extends the theoretical framework by proposing the idea of employees shifting their identification from the organization to the leader due to perceived organizational injustice. By uncovering the moderating role of perceived organizational justice in PsyCap transfer and the impact of leaders’ PsyCap on followers’ resilience, this research advances the understanding of these critical dynamics within organizations.

## 2 PsyCap and the cross-over effect

Employees with high levels of PsyCap have been highly studied in organizational psychology. Avey et al. (2011b) conducted a comprehensive meta-analysis supporting the positive impact of PsyCap on various employee outcomes, such as job satisfaction, commitment, organizational citizenship behavior (OCB), and overall well-being. Moreover, they observed that high PsyCap correlates with lower levels of cynicism, anxiety, stress, deviant behavior, and turnover intention. Several subsequent studies have further reinforced these findings by showcasing the beneficial relationship between PsyCap and a wide array of outcomes, including creative and innovative performance, job role effectiveness, productivity, career advancement, work engagement, happiness, reduced absenteeism, and sustained job satisfaction (Avey et al., 2008; Peterson et al., 2011; Rego et al., 2012; Luthans et al., 2013; Bouckenooghe et al., 2015; Huang and Luthans, 2015; Williams et al., 2015; Youssef-Morgan and Luthans, 2015; Baykal and Zehir, 2018; Du Plessis and Boshoff, 2018; Järlström et al., 2020; Karakitapoğlu-Aygün et al., 2020; Özsungur, 2020). Additionally, studies have demonstrated PsyCap’s negative association with turnover intention, stress, and depressive symptoms (Avey et al., 2009; Huang et al., 2023). Further exploration into PsyCap’s influence has uncovered its role as both a mediator and a moderator in various organizational contexts. It has been identified as a mediator in relationships between authentic leadership and work engagement, as well as between conveyed leader PsyCap and creative task performance (Avey et al., 2012; Du Plessis and Boshoff, 2018). Moreover, PsyCap moderates relationships such as leader-member exchange with performance and employee creativity (Wang et al., 2014; Kalyar et al., 2019). Especially in the context of today’s dynamic and uncertain organizational environments, the importance of resilient employees who can perform effectively despite challenges cannot be overstated. These resilient employees possess the capacity to adjust their functioning before, during, and after disruptions (Son et al., 2020).

The development and transfer of resilience and PsyCap within an organization are critical with leadership playing a pivotal role in this process (Avey et al., 2011a; Haar et al., 2014). Research illustrates that leaders with high PsyCap positively influences their followers’ PsyCap (Walumbwa et al., 2010; Chen, 2015). The impact of trickle-down leadership on PsyCap can occur through various mechanisms. For instance, leaders with high PsyCap are more inclined to set challenging goals and provide feedback that facilitates employee learning and growth (Luthans et al., 2007). Additionally, they may foster a positive work environment that supports employee well-being and reduces stress (Luthans and Youssef-Morgan, 2017). Furthermore, leaders exhibiting high PsyCap may display transformational leadership behaviors, such as inspiring and motivating their followers, leading to increased employee engagement and job satisfaction (King et al., 2015; Zhang et al., 2023). Therefore, the impact of trickle-down leadership on PsyCap can occur through both direct and indirect mechanism. This relationship is mediated by factors such as leader-member exchange, followers’ organizational identification, and moderated by follower self-esteem and team collectivism (Avey et al., 2012; Story et al., 2013; Xu et al., 2017; Chen et al., 2019). Evidence supports the existence of PsyCap cross-over (Walumbwa et al., 2010; Avey et al., 2011a; Chen, 2015), although only a limited number of studies have explored this trickle-down relationship. Initial research suggests that follower self-esteem moderates this relationship, with a more pronounced effect among individuals with lower self-esteem (Avey et al., 2012). Another identified moderator is collectivism, reflecting a dimension of team culture. In a highly collective culture, employees prioritize the interests of the team and the organization over their individual goals. Conversely, a low collective culture promotes self-interest and individual development. In such environments, employees are more likely to rely on the leader’s PsyCap for personal growth. Thus, the cross-over of PsyCap becomes more pronounced in contexts with lower team collectivism and weaker in contexts with higher team collectivism (Xu et al., 2017). However, the existing research provides limited insights and practical implications for enhancing followers’ PsyCap through leadership processes. The context in which the PsyCap trickle-down effect occurs remains relatively unknown, emphasizing the necessity to identify moderators influencing the significance of PsyCap in the workplace (Walumbwa et al., 2010; Avey et al., 2011b; Luthans and Youssef-Morgan, 2017; Xu et al., 2017). The identification of such moderators would offer insights into situations where PsyCap is particularly vital for business outcomes and aid in formulating effective HR strategies.

## 3 Theory background and hypothesis development

PsyCap and resilience are considered malleable resource that can be developed, albeit being more stable compared to emotions (Luthans et al., 2007). Research indicates that PsyCap can be enhanced through short training interventions highlighting its cognitive foundations (Luthans et al., 2006, 2007, 2010; Peterson et al., 2011). Similarly, resilience can be strengthened by the experiencing adversities and overcoming obstacles (Eve and Kangas, 2015). The explanation of this process is rooted in self-reflection, which involves a “metacognitive approach to learning allowing individuals to develop self-insight” (Crane et al., 2018). Social learning theory provides insights into how individuals acquire new skills and knowledge by observing credible and influential role models (Bandura, 1977). Within organizations, supervisors are often perceived as influential figures, with employees more inclined to imitate those of higher status and power (Mayer et al., 2009). To provide a comprehensive understanding of moderation in this model, integrating social identity theory is proposed. According to social identity theory, social identity encompasses cognitive identity processes (identification) and behavioral components that influence the expression of identities (Stryker and Serpe, 1994). In organizational contexts, identification is typically viewed as an individual’s alignment with the organization. However, this study adopts a broader interpretation, suggesting that subordinates aspire to resemble their leaders and possess similar qualities (Kelman, 1961; Ashforth and Mael, 1989). Through identification, individuals internalize the values, norms, attitudes, and behaviors of the group or another person (Ashforth and Mael, 1989). When an employee identifies with their leader, it’s likely that the subordinate’s PsyCap will mirror that of their leader, a phenomenon supported by Chen et al. (2017), emphasizing identification’s crucial role in mediating the PsyCap cross-over effect. The significance of identification with a leader intensifies when the leader is esteemed and respected by followers (Huang and Luthans, 2015). Conversely, a leader who exhibits hopefulness, positive expectations about the future, self-confidence, persistence toward goals, and resilience may be more appealing and credible to employees (Walumbwa et al., 2010). Consequently, a supervisor with a high level of PsyCap becomes a more convincing role model to emulate. Moreover, their increased attractiveness enhances the likelihood of followers identifying with them. Integrating these social theories suggests a link between followers’ PsyCap and their leader’s PsyCap. Building upon this, the following hypothesis is proposed:

*Hypothesis* 1: *The follower’s PsyCap is positively related to the leader’s PsyCap.*

Recently, age has become an important factor in organizational research due to aging workforces, and as a specific demographic variable, it has emerged as significant. Age moderates many relationships, such as the relationship between procedural justice and turnover (Bal et al., 2011), the relationship between perception of procedural justice and long sickness absences (Tenhiälä et al., 2013), the relationship between HRM and work engagement (Gostautaite et al., 2019), and the relationship between task-specific self-efficacy beliefs and effort expenditure in organizational teamwork (Gärtner and Hertel, 2020). Furthermore, the relationship between congruency in implicit and explicit motives and job satisfaction is stronger for older workers compared to younger ones (Thielgen et al., 2015).

In the context of PsyCap transfer, the leader’s role, drawing from social learning theory (Bandura, 1977) and social identity theory (Kelman, 1961; Ashforth and Mael, 1989), is deemed crucial. Older leaders are commonly perceived to possess greater experience, wisdom, responsibility, and dependability compared to their younger counterparts (Spisak et al., 2014). These qualities make them more appealing as role models for identification and learning purposes, suggesting that an older leader might be more effective in PsyCap modeling than a younger supervisor.

Traditionally, career timetable theory suggests that older, more experienced individuals should manage younger ones (Tsui and O’Reilly, 1989; Shore et al., 2003; Collins et al., 2009; Fritzsche and Baz, 2017). However, when a younger leader supervises older subordinates, it goes against this norm (Perry et al., 1999). People naturally compare their efforts and results with those of others (Festinger, 1954). If this comparison involves a young leader, it may lead to rejection and devaluation. Subordinates may perceive the difference in status as unjustified and unfair, resulting in the younger leader not receiving the same level of respect as an older leader. Employees who do not accept the authority of their supervisor may not identify with the leader, and the younger supervisor may struggle to become a role model. Consequently, compared to an older leader, a younger leader will have a weaker influence on employees, and the PsyCap cross-over effect will be diminished. The findings from this investigation could be a contribution to the ongoing age-related debate. Similar to previous studies, even in the context of PsyCap trickle-down, age appears to influence the main relationship.

*Hypothesis* 2: *Leaders’ age moderates the relationship between leaders’ and followers’ PsyCap such that the relationship is stronger for dyads with older leaders.*

In the workplace, employees who perceive fair treatment have confidence in their organization’s just compensation for their efforts on challenging tasks, viewing the organization as supportive (Wayne et al., 2002). This perception leads to a belief in their control over their interaction with the organization and fosters a higher level of efficacy. Just organizational environments motivate employees to excel and foster stronger commitment (Masterson et al., 2000; Colquitt et al., 2013), consequently enhancing their persistence in confronting problems and failures. An employee with high level of POJ anticipates fair incentives distribution within the organization and holds positive attributions about their role and future within the entity. They are less likely to consider leaving the organization (Skarlicki and Folger, 1997), satisfied with their job (Moorman, 1991), optimistic, and hopeful about the future (Luthans et al., 2007). Moreover, POJ also contributes to the organizational climate (Aquino et al., 2006) and is linked to the perceived ethical climate and moral standards (Castro-González et al., 2019). Considering that the psychological climate promotes employees’ PsyCap (Munyaka et al., 2017), I support the argument that the effect of POJ may be associated with a positive climate that fosters employees’ PsyCap. A positive, just, and predictable workplace climate nurtures employees’ optimism and efficacy, encouraging a hopeful and persistent attitude in their work. In this context, the hypothesis suggests a positive relationship between POJ and employees’ PsyCap:

*Hypothesis* 3: *There is a positive relationship between POJ and employees’ PsyCap.*

Previous research emphasizes the influential role of POJ in shaping employees’ perceptions within the organization and its potential to moderate the impact of various organizational factors on employee behavior. Empirical studies have demonstrated that POJ moderates the relationship between the human factor and the perception of organizational effectiveness (Acquaah and Tukamushaba, 2015), the link between emotional exhaustion and organizational loyalty (Hur et al., 2014), and the relationship between political skills and career success (Lu and Guy, 2018). Moreover, higher-than-average POJ weakens the impact of job insecurity on job performance (Schumacher et al., 2020). Additionally, POJ compensates for deficiencies in leadership or human resource management (HRM) and enhances various positive employee outcomes, such as facilitating the development of employees’ PsyCap, even when the followers’ PsyCap is low (Abas et al., 2018; Wei et al., 2020). The perception of higher POJ weakens the existing negative relationship between despotic leadership, narcissism, and work meaningfulness (Kayani et al., 2020). On the other hand, POJ decreases the impact of leader and HRM practices on follower behaviors, attitudes, and emotions (Abas et al., 2018), as shown in the relationship between abusive supervision and knowledge sharing mediated by emotional exhaustion, where the mediated relationship is stronger under low POJ compared to high POJ (Lee et al., 2018). POJ also plays a similar role in the relationship between psychological empowerment and voice behavior (Wei et al., 2020). The literature suggests that POJ moderates the relationship between leadership and employee outcomes in diverse ways, contingent upon specific employee outcomes and contextual factors. Integrating existing literature on POJ’s complex role in the leadership-employee outcomes relationship infers that POJ may foster the development of employees’ PsyCap, particularly when followers’ PsyCap is low. When employees perceive organizational injustice, they might withdraw identification with the organization and pivot toward identifying more with the leader instead, relying more on the leader and benefiting from the trickle-down effect. Therefore, the leader as a source of employees’ PsyCap might be more meaningful and effective in the case of organizational deficiencies. Considering these aspects, the fourth hypothesis posits that POJ moderates the relationship between leaders’ and followers’ PsyCap, proposing a link between organizational justice perception and the PsyCap trickle-down effect.

*Hypothesis* 4: *POJ moderates the relationship between leaders’ and followers’ PsyCap, such that the relationship is stronger when POJ is lower rather than higher.*

The moderation effect on resilience might be more pronounced than on overall PsyCap. Adversity and crises can potentially fortify resilience by providing experiences that facilitate its development (Tedeschi and Calhoun, 1995; Crane et al., 2018). According to the informative social influence theory, we learn how to react and cope with unexpected events from those around us (Deutsch and Gerard, 1955). In times of uncertainty or adversity we tend to mimic the supervisor’s behavior. Similarly, drawing from social learning theory, an employee with low POJ tends to rely more on the leader as a role model, extensively learning through a trickle-down process.

Consequently, when the leader demonstrates persistence in the face of failure, the follower’s resilience is likely to be reinforced. Therefore, it can be inferred that in situations of organizational injustice, the trickle-down effect on followers’ resilience is more robust compared to instances where POJ is high.

*Hypothesis* 4a. *POJ moderates the relationship between leaders’ PsyCap and followers’ resilience such that the relationship is stronger when POJ is lower rather than higher.*

**FIGURE 1:**
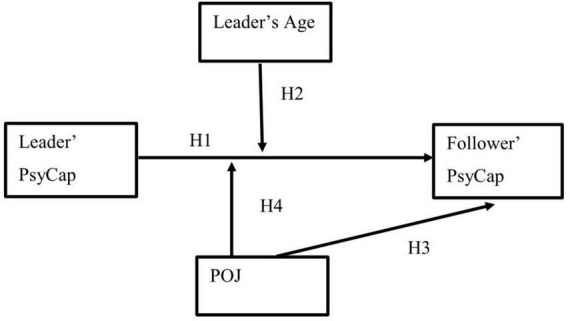
The hypothesized model.

The theoretical model of this study is summarized in Figure 1.

## 4 Methods

### 4.1 Sample and procedure

To test the hypotheses, a cross-sectional study was conducted on a randomly selected sample of 406 enterprises and 812 respondents. Leader-follower dyads were recruited for the study, with one dyad per organization, to ensure of independence among dyads in the sample. This would prevent the use of two-level multilevel regression analysis and introduce a third level of analysis. Recruitment of dyads began with the follower, followed by an invitation to the leader to participate. Data from complete dyads were exclusively considered for further analysis.

Data collection took place in Poland in April 2018, facilitated by a contracted company using the CAPI technique. Invitations to participate were emailed to 1200 organizations randomly chosen from the public register of companies, yielding an approximate response rate of 34%. The selected organizations represented various industries and sizes, excluding small enterprises (up to 50 employees). Screening of the sample sought to exclude small-sized enterprises and non-managerial positions among respondents. All data were collected simultaneously, with efforts made to minimize the time gap between data collection from the leader and the follower. The average age of the leader was 44.5°years, and the average age of the follower was 35.5°years. Women comprised 50.7% of leaders and 49.8% of followers. The distribution of businesses by industry was as follows: 32.3% in manufacturing (131 companies), 39.2% in retail and gastronomy (160 enterprises), 20.9% in business-oriented services (84 companies), and 31 companies in consumer-oriented services, constituting 7.6%. The slight bias toward the manufacturing industry is due to the exclusion of small enterprises. The companies had been operating for an average of 21°years and employed an average of 458 employees. To test the inattentiveness of respondents and ensure the validity of the collected data, intraindividual response variability was calculated. The mean within-person standard deviation of raw scores varied from 0.53 to 0.68 (on a 7-point scale), depending on the item range and respondent type (leader/follower). This suggests an average intraindividual response variability and, therefore, the absence of both overly consistent or random responding (Hong et al., 2020).

### 4.2 Measurements

PsyCap was assessed using Luthans et al. (2007) measurement comprising of 24 items. An example item is: “I usually take stressful things at work in stride.” Cronbach’s coefficients were 0.946 for leaders and 0.952 for followers. Confirmatory factor analysis indicated a good fit for both leaders (RMSEA = 0.056, CFI = 0.952, TLI = 0.946, SRMR = 0.035) and followers (RMSEA = 0.065, CFI = 0.939, TLI = 0.932, SRMR = 0.037). Resilience and each other dimension of PsyCap is measured by 6 items.

Perceived organizational justice (POJ) was measured using Colquitt’s (2001) scale with 18 items. The questions referred to performance appraisal, and an example item is: “Does your outcome reflect the effort you have put into your work?” POJ was evaluated for followers, yielding an alpha coefficient of 0.973. Confirmatory factor analysis revealed an acceptable fit (RMSEA = 0.083, CFI = 0.948, TLI = 0.939, SRMR = 0.028).

Harman’s Single Factor Test was executed to check for common method bias. The variance explained by the single extracted factor was 42.25%, indicating that common method bias did not significantly impact the results. PsyCap and POJ were measured using single measurements, which were aggregated from the dimensions following the commonly used approach (Avey et al., 2011b). The summary of the scales used is presented in Table 1.

**TABLE 1:** Summary of the used scales.

Measure	*Psychological capital (leader)*	*Psychological capital (follower)*	*Perceived organizational justice*
Number of items	24	24	18
Measurement scale	7-point	7-point	7-point
Cronbach’s alpha	0.946	0.952	0.973
RMSEA	0.056	0.065	0.083
CFI	0.952	0.939	0.948
TLI	0.946	0.932	0.939
SRMR	0.035	0.037	0.028

To investigate alternative explanations for observed relationships, several variables were checked for their significance on the follower’s PsyCap: follower’s age (Aliyev and Tunc, 2015), tenure (in years) (Avey et al., 2010), gender (Caza et al., 2010), as well as age and size of the enterprise (Zhang et al., 2020). Gender was coded as 1 for women and 0 for men. Age and size of the enterprises were included in the calculations as natural logarithms due to the high skewness of the distribution. The descriptive statistics of the variables are presented in Table 2. The analysis was conducted as a multilevel dyadic regression analysis (Kenny et al., 2020). The actor-partner interdependence model (e.g., Brown et al., 2021) was applied to capture the effect of the leader’s PsyCap on the follower’s PsyCap. As the dyads in the study were based on a distinguishable factor (leader’s supervision over the follower), the data were restructured to convert a dyad into a single unit of analysis. OLS regression was then performed to verify the results and enhance their robustness. Findings from both analyses indicated the same direction and level of significance. Descriptive statistics for the variables are detailed in Table 2.

**TABLE 2:** Descriptive statistics of variables.

Variable	N		Mean	Median	SD	Min	Max
	**Valid**	**Missing**					
Leader’s PsyCap.	406	0	5,5056	5,4792	0,75832	3,83	7,00
Follower’s PsyCap	406	0	5,2415	5,1875	0,79405	3,21	7,00
Justice	406	0	5,1426	5,2188	0,92045	2,56	7,00
Leader age	406	0	44,5123	43,0000	9,34674	25,00	71,00
Follower age	406	0	35,4581	33,0000	8,88539	19,00	62,00
Leader tenure	406	0	12,1626	10,0000	7,94204	1,00	50,00
Follower tenure	406	0	6,3966	5,0000	6,19700	1,00	39,00
Leader’s gender (0-male, 1-female)	406	0	0,5074	1,0000	0,50056	0,00	1,00
Follower’s gender (0-male, 1-female)	406	0	0,4975	0,0000	0,50061	0,00	1,00

## 5 Results

At the outset of the statistical analysis, a correlation analysis was performed to examine the relationships between the variables. Table 3 presents the descriptive statistics (means and standard deviations) and Pearson’s correlation analysis.

**TABLE 3:** Descriptive statistics and correlations between variables.

No.	Variable	Mean	SD	1	2	3	4	5	6	7	8
1	Leader’s PsyCap	5.506	0.758								
2	Follower’s PsyCap	5.241	0.794	0.719**							
3	POJ	5.143	0.920	0.438**	0.584**						
4	Leader’s age	44.512	9.347	0.020	0.030	−0.002					
5	Follower’s age	35.458	8.885	0.050	0.066	0.035	0.414**				
6	Follower’s tenure	6.397	6.197	0.096	0.062	0.047	0.325**	0.701**			
7	Follower’s gender	0.498	0.501	−0.018	−0.003	−0.113*	−0.060	−0.107*	−0.041		
8	Business age (ln)	2.847	0.647	0.068	0.012	−0.047	0.211**	0.194**	0.314**	0.008	
9	Business size (ln)	4.728	0.992	0.066	0.061	0.030	−0.086	0.009	0.021	0.015	0.196**

*p < 0.05,

**p < 0.01.

The results of the correlation analysis suggest potential relationships between the variables, particularly between the independent variable (leader’s PsyCap) and dependent variable (follower’s PsyCap). The correlation coefficients also indicate the presence of multicollinearity. To assess multicollinearity, a Variance Inflation Factor (VIF) analysis was conducted. The highest VIF values were observed for leader’s age and follower’s tenure. However, the highest VIF values were within 2.8, indicating that multicollinearity was not inflating the analysis results.

Six multilevel dyadic regression models were tested for followers’ PsyCap (Table 4). Robustness check with standardized variables was conducted and the results hold in that version (Table 5). Six multilevel dyadic regression models were tested for followers’ resilience (Table 6). In the first model (Model 1), only the control variables were included. The second model (Model 2) added the independent variable, follower’s PsyCap. The third model (Model 3) included potential moderators: leader’s age and POJ. To separately examine the moderations as they may be conditional upon one another (Hayes, 2017), each interaction term was individually added (leader’s age in Model 4, POJ in Model 5), and together in one model (Model 6). For all models, an analysis of the R2 coefficient was conducted, and for Models 2 to 6 underwent a likelihood ratio test to compare them to the preceding models. Models 4 to 6 were compared to Model 3. The results of the analysis with the original and standardized variables are presented in Tables 4, 5, respectively.

**TABLE 4:** The results of the multilevel dyadic regression analyses (unstandardized variables) for follower PsyCap.

	(1)	(2)	(3)	(4)	(5)	(6)
Leader’s PsyCap		0.755***	0.600***	0.096	1.485***	0.984***
		(0.037)	(0.037)	(0.162)	(0.183)	(0.240)
Leader’s age			0.001	−0.060**	0.002	−0.058**
			(0.003)	(0.020)	(0.003)	(0.019)
Leader’s PsyCap x Leader’s age				0.011**		0.011**
				(0.003)		(0.003)
POJ			0.291***	0.289***	1.363***	1.348***
			(0.030)	(0.030)	(0.220)	(0.217)
Leader’s PsyCap x POJ					−0.183***	−0.181***
					(0.037)	(0.037)
Female	0.004	0.025	0.081	0.076	0.109*	0.105*
	(0.080)	(0.055)	(0.050)	(0.050)	(0.049)	(0.049)
Follower’s age	0.004	0.006	0.006	0.005	0.008+	0.007+
	(0.006)	(0.004)	(0.004)	(0.004)	(0.004)	(0.004)
Tenure	0.005	−0.005	−0.007	−0.006	−0.010+	−0.009
	(0.009)	(0.006)	(0.006)	(0.006)	(0.006)	(0.006)
Age of company (ln)	−0.025	−0.051	−0.017	−0.023	−0.018	−0.024
	(0.066)	(0.046)	(0.042)	(0.042)	(0.041)	(0.040)
Size of company (ln)	0.051	0.017	0.014	0.018	0.023	0.027
	(0.041)	(0.028)	(0.026)	(0.026)	(0.025)	(0.025)
Constant	4.901***	0.953***	0.162	2.988**	−5.129***	−2.319
	(0.308)	(0.287)	(0.285)	(0.930)	(1.110)	(1.407)
Dyads	406	406	406	406	406	406
R^2^	0.009	0.521	0.611	0.621	0.634	0.643
		0.512	0.090	0.010	0.023	0.032
LR test		295.210***	84.720***	10.300**	24.120***	34.450***
		(1)	(2)	(3)	(3)	(3)

*p < 0.05,

**p < 0.01,

***p < 0.001.

**TABLE 5:** Robustness check: the results of the multilevel dyadic regression analyses (standardized variables) for follower PsyCap.

	(1)	(2)	(3)	(4)	(5)	(6)
Leader’s PsyCap		0.721***	0.573***	0.518***	0.565***	0.511***
		(0.035)	(0.035)	(0.036)	(0.035)	(0.036)
Leader’s age			0.017	0.028	0.012	0.023
			(0.035)	(0.034)	(0.035)	(0.034)
Leader’s PsyCap x Leader’s age					0.100**	0.097**
					(0.031)	(0.030)
POJ			0.338***	0.410***	0.335***	0.406***
			(0.035)	(0.037)	(0.035)	(0.037)
Leader’s PsyCap x POJ				−0.161***		−0.159***
				(0.033)		(0.032)
Female	0.005	0.031	0.102	0.138*	0.096	0.066*
	(0.100)	(0.070)	(0.063)	(0.062)	(0.063)	(0.031)
Follower’s age	0.044	0.069	0.063	0.087+	0.055	0.079+
	(0.070)	(0.049)	(0.046)	(0.045)	(0.046)	(0.045)
Tenure	0.037	−0.041	−0.052	−0.079+	−0.046	−0.072
	(0.072)	(0.050)	(0.046)	(0.045)	(0.045)	(0.044)
Age of company (ln)	−0.021	−0.042	−0.014	−0.015	−0.019	−0.020
	(0.054)	(0.037)	(0.034)	(0.033)	(0.034)	(0.033)
Size of company (ln)	0.064	0.022	0.017	0.029	0.023	0.034
	(0.051)	(0.035)	(0.032)	(0.031)	(0.032)	(0.031)
Constant	−0.003	−0.016	−0.051	0.002	−0.050	0.067*
	(0.070)	(0.049)	(0.044)	(0.044)	(0.044)	(0.033)
Dyads	406	406	406	406	406	406
R^2^	0.009	0.521	0.611	0.621	0.634	0.643
**δ R^2^**						
LR test		295.210***	84.720***	10.300**	24.120***	34.450***
		(1)	(2)	(3)	(3)	(3)

*p < 0.05,

**p < 0.01,

***p < 0.001.

**TABLE 6:** The results of the multilevel dyadic regression analyses (unstandardized variables) for follower resilience.

	(1)	(2)	(3)	(4)	(5)	(6)
Leader’s PsyCap		0.751***	0.577***	0.103	1.678***	1.211***
		(0.042)	(0.043)	(0.189)	(0.212)	(0.278)
Leader’s age			−0.004	−0.062**	−0.003	−0.059**
			(0.003)	(0.023)	(0.003)	(0.022)
Leader’s PsyCap x Leader’s age				0.010*		0.010*
				(0.004)		(0.004)
POJ			0.326***	0.324***	1.659***	1.645***
			(0.035)	(0.035)	(0.254)	(0.252)
Leader’s PsyCap x POJ					−0.228***	−0.226***
					(0.043)	(0.043)
Female	−0.069	−0.049	0.012	0.008	0.048	0.043
	(0.085)	(0.064)	(0.058)	(0.058)	(0.057)	(0.057)
Follower’s Age	0.003	0.005	0.007	0.006	0.009*	0.009+
	(0.007)	(0.005)	(0.005)	(0.005)	(0.005)	(0.005)
Tenure	0.005	−0.005	−0.006	−0.005	−0.010	−0.010
	(0.010)	(0.007)	(0.007)	(0.007)	(0.007)	(0.007)
Age of company (ln)	−0.021	−0.047	0.003	−0.002	0.003	−0.003
	(0.071)	(0.053)	(0.049)	(0.048)	(0.047)	(0.047)
Size of company (ln)	0.050	0.016	0.006	0.010	0.017	0.022
	(0.044)	(0.033)	(0.030)	(0.030)	(0.029)	(0.029)
Constant	5.107***	1.181***	0.460	3.115**	−6.119***	−3.503*
	(0.331)	(0.332)	(0.331)	(1.084)	(1.283)	(1.633)
Dyads	406	406	406	406	406	406
R^2^	0.009	0.447	0.546	0.554	0.577	0.583
		0.438	0.108	0.008	0.023	0.006
LR test		237.04***	80.51***	27.80***	6.72***	34.48***
		(1)	(2)	(3)	(3)	(3)

*p < 0.05,

**p < 0.01,

***p < 0.001.

The decision to accept or reject specific hypotheses was based on the adjusted R2 coefficient and the likelihood ratio test. The results demonstrate a strong positive relationship between leader’s and follower’s PsyCap (Model 2, *B* = 0.755, *p* < 0.001). Furthermore, the adjusted R2 coefficient for Model 2 was 0.521, indicating a high explanatory power of the model and a substantial effect of leader’s PsyCap on follower’s PsyCap. This result was confirmed by the likelihood ratio test. Therefore, hypothesis 1 can be accepted.

**FIGURE 2:**
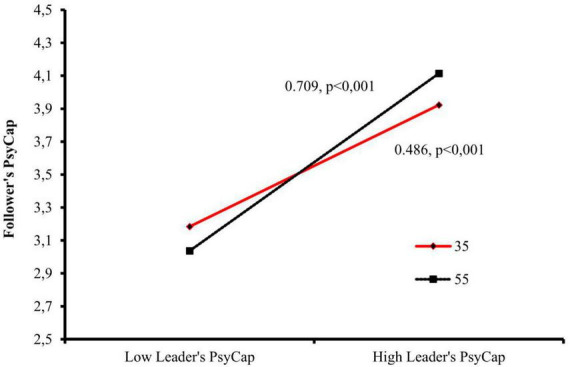
Moderation of the relationship between leader’s PsyCap and follower’s PsyCap by leader’s age.

The interaction terms between leader’s PsyCap and leader’s age were statistically significant (Model 4, *B* = 0.011, *p* = 0.002; Model 6, *B* = 0.011, *p* = 0.002), indicating a moderation effect for followers’ PsyCap as dependent variable. Additionally, the inclusion of the interaction terms significantly improved the model fit (LR test = 10.3, *p* = 0.001), confirming hypothesis H2. Figure 2 graphically presents this moderation effect.

In the graphical representation, two values of leader’s age are depicted - 35 and 55°years, chosen as meaningful values closest to one standard deviation above and below the mean age of the leaders (35.15 and 53.85). These values did not affect the moderation results. For leader’s PsyCap, values were selected based on established convention (+/− 1SD: 4.74; 6.26).

The moderation analysis demonstrates that leader’s age moderates the relationship between leader’s and follower’s PsyCap. The relationship is stronger for older leaders. A simple slope test indicates that for leaders at the age of 35, the estimated regression coefficient for leader’s PsyCap is 0.486, while for leaders at the age of 55, it is 0.709. Therefore, the relationship is significant for all leaders, but stronger for older leaders than for younger leaders.

**FIGURE 3:**
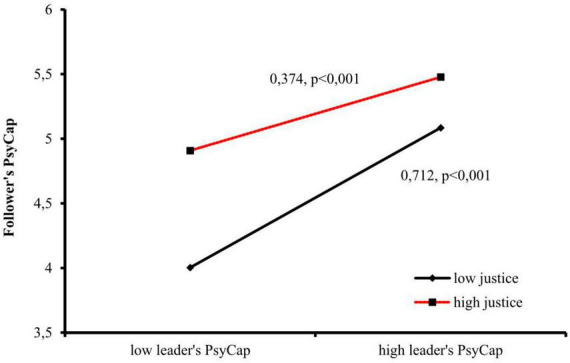
Moderation of the relationship between leader’s PsyCap and follower’s PsyCap by POJ.

The interaction terms between leader’s PsyCap and POJ were statistically significant for the dependent variable followers’ PsyCap (Model 5, *B* = −0.183, *p* < 0.001; Model 6, *B* = −0.181, *p* < 0.001), indicating a moderation effect. Moreover, the inclusion of the interaction terms significantly improved the model fit (LR test = 24.12, *p* < 0.001), confirming hypothesis H4. Figure 3 graphically presents this moderation effect.

Perceived organizational justice (POJ) supports the follower’s PsyCap and moderates the effect of leader’s PsyCap, particularly in low-POJ conditions where the effect is stronger. This suggests that a leader with high PsyCap is especially beneficial for the follower in organizations with low POJ. A simple slope test indicates that in low POJ (−1SD), the estimated coefficient of leader’s PsyCap is 0.712, while in a high POJ environment, it is 0.374. Notably, follower’s PsyCap reaches high levels in high-POJ conditions, and a combination of high POJ and high leader;s PsyCap elevates follower’s PsyCap to the highest level.

The interaction terms between leader’s PsyCap and POJ were also statistically significant for followers’ resilience (Model 5, *B* = −0.228, *p* < 0.001; Model 6, *B* = −0.226, *p* < 0.001), indicating a moderation effect, confirming hypothesis H4 A.

To ensure the robustness of the results, several alternative explanations, including authentic leadership, fit between leader and follower, trust, and frequency of communication, were examined. The results remained consistent across all analyses. Additionally, the final model was replicated using separate dimensions of POJ and PsyCap, and the direction and significance of the relationships remained unchanged. An additional analysis considering the age difference between the leader and follower did not yield significant results, and the remaining findings remained consistent.

## 6 Discussion

Previous research identifies three primary drivers of PsyCap: individual differences, job characteristics and leadership. Earlier analyses indicate that supervision is a pivotal antecedent, explaining 32% of the variance of employee PsyCap (Avey, 2014). In this study, the explanatory power of leaders’ PsyCap is R^2^ = 0.521, marking it as a significant antecedent compared to R^2^ = 0.009 in the model with controls only. Therefore, the leader’s PsyCap explains approximately 50% of the followers’ PsyCap. By confirming the positive influence of a leader’s PsyCap on a follower’s PsyCap, this research empirically validates previous findings on the cross-over effect (Walumbwa et al., 2010; Story et al., 2013; Chen et al., 2017). Moreover, the substantial explanatory power of this study contributes significantly to the ongoing debate on the trickle-down effect of PsyCap.

High levels of both employees’ PsyCap and their resilience are desirable. Thus, understanding the conditions for the cross-over effect and the relationship between leaders’ PsyCap and followers’ resilience leads to questions about potential moderators. Prior studies have identified follower self-esteem (Avey et al., 2012) and team collectivism (Xu et al., 2017) as moderators in the relationship between leaders and followers’ PsyCap and resilience. However, these investigations do not present the complete picture, leaving room for further exploration. Addressing a gap in the PsyCap literature, this study hypothesized that POJ and leader’s age act as moderators influencing the strength of the trickle-down effect. The impact of POJ on employees’ PsyCap was also examined. The research outcomes demonstrated the moderating role of both leaders’ age and POJ.

The relationship between leaders and followers’ PsyCap is more robust for dyads with older leaders. This aligns with previous research on older leaders and career timetable theory (Shore et al., 2003; Fritzsche and Baz, 2017). An older supervisor is more readily accepted as a leader and is perceived as a role model from whom employees learn more compared to a younger supervisor. The PsyCap of an older leader might be viewed as more attractive, valuable, and associated with wisdom. Conversely, a younger supervisor may lack the legitimacy to hold a privileged position, resulting in reduced identification with the leader and resistance to learning from them. POJ moderates both relationships of the leaders’ PsyCap as the independent variable with followers’ PsyCap and followers’ resilience. The moderation effect is stronger in the case of resilience than for the overall followers’ PsyCap. This implies that when there is a lack of justice, leaders’ PsyCap has a greater impact on the followers’ ability of bouncing back from the setbacks than on the followers’ overall PsyCap.

The results of this study affirm the positive impact of POJ on PsyCap, highlighting the valuable role of POJ in organizations. Previous research has identified POJ an important antecedent and boundary condition for various positive organizational outcomes and employee behaviors, while also being negatively associated with undesirable behaviors. The moderating effect of POJ in relation to PsyCap and resilience extenda the relevance of justice theory, further enriching our understanding of POJ. These results also contribute to the discussion on the advantages of older age, particularly in leadership.

The PsyCap literature holds an ongoing debate regarding the conceptual framework of the PsyCap cross-over effect. Some researchers view the leader-follower exchange as the primary transfer rule, emphasizing the trade value of PsyCap (Story et al., 2013; Chen et al., 2019). Others base their arguments on contagion theory (Walumbwa et al., 2010; Story et al., 2013), likening PsyCap to emotions. In contrast, this study challenges these assumptions and adopts social learning theory and social identity theory (Chen et al., 2017) to elucidate the moderating role of POJ and leaders’ age. A similar approach was employed in a recent study by Xu et al. (2017), which also emphasized the importance of resources like PsyCap through conservation resource theory. When compared with other studies on the trickle-down mechanism, it becomes evident that the transfer process from supervisors to followers is characteristic of soft resources and attitudes expressed through specific behaviors. Behavioral integrity (Simons et al., 2007; He and Feng, 2018), psychological safety (Lance and Tupper, 2018), ethical leadership (Mayer et al., 2009), abusive behaviors (Mawritz et al., 2012; Rice et al., 2021), authenticity (Gardner et al., 2005), and perception of interpersonal justice (Wo et al., 2015) share similarities with PsyCap in terms of malleability and developability. These individual characteristics are transferable, and the theoretical frameworks adopted in these studies to explain the effects also refer to social learning theories.

This study contributes to the scholarly discussion on age in management. There is extensive research on the implications of age on workplace behavior and management, along with age-related stereotypes and potential discrimination (Rudolph and Zacher, 2015). Some studies highlight the advantages of younger employees, such as flexibility or higher learning orientations (e.g., Gärtner and Hertel, 2020), while others argue that these characteristics are mere age stereotypes (e.g., Ng and Feldman, 2012; Spisak et al., 2014). Conversely, some studies show that older employees possess the advantage of higher emotional stability (e.g., Walter and Scheibe, 2013). This research demonstrates the benefits of leaders’ older age as a boundary condition that facilitates the transmission of PsyCap. The findings of this study also expand the leadership literature, which often focuses on specific leadership styles. These findings provide an important step in uncovering the explanatory mechanisms for how PsyCap is transferred in a trickle-down direction.

## 7 Conclusions and managerial implications

The main research question of this study was revolved around identifying when the PsyCap cross-over effect is stronger and when the impact of leader PsyCap on followers’ resilience is more significant. This investigation aimed to address a research gap by exploring how organizations could foster positive psychological resources, such as resilience and PsyCap, within their workforce, shedding light on the role of leaders’ PsyCap. It was hypothesized that followers’ PsyCap correlates positively with leaders’ PsyCap along with a positive relationship between POJ and employees’ PsyCap. Two moderators were proposed: leaders’ age moderating the relationship between leaders’ and followers’ PsyCap to be stronger for dyads with older leaders; and perceived justice, moderating the relationship between leaders’ PsyCap and followers’ PsyCap, and between leaders’ PsyCap and followers’ resilience, particularly stronger when POJ is lower.

This cross-sectional study was conducted with a randomly selected sample of 812 respondents, comprising 406 leader-follower dyads. The research findings supported all hypotheses, emphasizing that the moderation of POJ holds more significance for followers’ resilience than for followers’ overall PsyCap.

Future studies employing longitudinal and experimental designs are recommended to ascertain causality and explore alternative causal paths. Further research on PsyCap should delve deeper into boundary conditions and antecedents, encompassing personality traits, perceived organizational support, trust, calling, thriving, the leader-follower relationship, and cultural differences. The outcomes of this study contribute significantly to the leadership literature and the ongoing discourse on the advantages of older age among managers. They validate that experienced and older leaders serve as better role models for PsyCap.

One of the primary responsibilities of leaders today is to foster the enhancement of their followers’ working skills and positive psychological attributes (Chen et al., 2019). Given that PsyCap and resilience can be cultivated and altered (Luthans et al., 2006, 2010), these findings hold practical implications for human resource development and leadership. Supervisors should be adept at nurturing employees’ PsyCap and resilience through behaviors that exemplify optimism, hope, efficacy, and resilience. For example, when encountering obstacles at work, a manager who openly displays hope and optimism effectively communicates messages about their own PsyCap and the expected attitude of their staff in similar situations. This can influence employees’ perceptions of PsyCap in several ways. Firstly, employees witnessing their supervisor exhibiting a high level of PsyCap are likely to feel prompted to engage similarly. This aligns with social learning theory, as employees observe and learn workplace-appropriate behaviors and attitudes from their supervisors (Bandura, 1977). Secondly, an optimistic and resilient leader may be perceived more positively, leading to a stronger employee identification with the leader. Thirdly, during times of crisis and uncertainty, a resilient leader becomes a learning model for handling failure, which aids in fostering resilience in followers.

These results not only deepen our comprehension of the influential mechanisms of leaders’ PsyCap on their followers but also amplify our understanding of the positive impact of leaders’ age and the ambivalent role of POJ. Consequently, these findings are pertinent for employee selection and development. Teams led by older supervisors might exhibit a more effective PsyCap cross-over effect compared to those led by younger leaders. By aligning teams based on this criterion, the trickle-down of PsyCap could be augmented. Hence, PsyCap might serve as an evaluative criterion for leadership positions, providing employees with a role model and enhancing their own PsyCap. Managers should recognize the resources associated with age and encourage the transfer of PsyCap by supporting senior employees. Conversations about diversity and inclusiveness should encompass aging employees and their organizational value since their age supports the development of employees’ PsyCap. PsyCap can also compensate for perceived low levels of POJ. By acknowledging a deficit in POJ, a leader can stimulate employees’ PsyCap through their behaviors.

## 8 Limitations and future research

This study possesses several limitations worth noting. The research design being cross-sectional lacks the ability to establish causation, and it is susceptible to common method bias. The direction of impact is assumed theoretically and build upon prior research in the field. Therefore, it is highly recommended to undertake the longitudinal and experimental research to enable causal inference and validate alternative causal pathways. Additionally, the findings might hold alternative explanations, possibly reflecting the paradox described by Cameron (2008). On one hand, people tend to incline toward positive tendencies fostering a propensity for positive change in human systems. On the other hand, individuals might react more intensely to negative stimuli, implying that a low level of POJ might exert a stronger influence in deriving PsyCap from a leader. Conversely, a high level of POJ could directly stimulate employee PsyCap.

Future research on PsyCap should delve into exploring boundary conditions and antecedents- such as personality traits, perceived organizational support, trust, calling, and thriving. One potential moderator could be the type of transgression, thereby investigating how different types of transgressions influence the relationship between leader and follower PsyCap. Considering various types of leaders’ transgressions, recoverable and unrecoverable transgressions might affect this relationship differently (Epitropaki et al., 2020). Task-focused transgressions may have a less negative moderating effect on the transfer of PsyCap than person-focused transgressions.

An examination of followership might offer insight into when the influence of a leader’s PsyCap is most significant. The transfer of PsyCap from leader to follower could also hinge on how a leader enters into a relationship. Bastardoza and Van Vugt (2019) suggest three key strategies for becoming a leader: dominance, prestige, and charisma. In dominance-based relationships, followers are often passive, conformist, and less engaged. On the contrary, the other two strategies create opportunities for relationships in which followers willingly follow their leader, paving the way for the leader to serve as a role model. It can be posited that a dominant leader might be less likely to elevate followers’ PsyCap. Cultural differences, as underscored by Luthans and Youssef-Morgan (2017), should also be a focal point for future research, not merely as control variables but as significant boundary conditions. A deeper exploration in this area is essential for comprehending the transfer of PsyCap across cultures, vital for devising effective human resource development strategies in a global context.

Furthermore, leaders’ domain-specific PsyCap, like health PsyCap or relationship PsyCap (Luthans et al., 2013), might not only impact followers’ PsyCap but also influence their health strategies, hazardous behaviors, work-life balance, or conflict management. Therefore, delving further into this direction warrants investigation. The relationship between POJ and PsyCap is likely indirect, and potential mediators like employee commitment or other individual characteristics should be taken into account. Moreover, the PsyCap-POJ relationship might be moderated by organizational climate, particularly in a supportive climate, prompting the need for further investigation.

## Data availability statement

The raw data supporting the conclusions of this article will be made available by the authors, without undue reservation.

## Author contributions

MG-Z: Writing – original draft.
